# UMOD and you! Explaining a rare disease diagnosis

**DOI:** 10.1007/s44162-022-00005-4

**Published:** 2022-12-07

**Authors:** Holly Mabillard, Eric Olinger, John A. Sayer

**Affiliations:** 1 Translational and Clinical Research Institute, Faculty of Medical Sciences, Newcastle University, Central Parkway, Newcastle upon Tyne, NE1 3BZ UK; 2 Renal Services, Newcastle Upon Tyne Hospitals NHS Foundation Trust, Newcastle upon Tyne, NE7 7DN UK; 3 NIHR Newcastle Biomedical Research Centre, Newcastle upon Tyne, NE4 5PL UK

## Abstract

The precise molecular genetic diagnosis of a rare inherited disease is nearly always a prolonged odyssey. Fortunately, modern molecular testing strategies are allowing more diagnoses to be made. There are many different rare inherited kidney diseases and both the genetic heterogeneity of these conditions and the clinical diversity often leads to confusing nomenclature. Autosomal dominant tubulointerstitial kidney disease (ADTKD) is an example of this. ADTKD, an inherited kidney disease that leads to worsening of kidney function over time, often culminating in end stage kidney disease, accounting for around 2% of this cohort. *UMOD* is the most common gene implicated in this disorder but there are at least 6 subtypes. At present, there are no specific treatments for ADTKD. Here, we review the current understanding of this condition and provide patient-centred information to allow conceptual understanding of this disease to allow better recognition, diagnosis and management.

## Introduction

Autosomal dominant tubulointerstitial kidney disease (ADTKD) is an inherited kidney disease that leads to worsening of kidney function over time. As patients get older, they may require dialysis or kidney transplantation and the age of onset of kidney failure varies within and between families (ranging from 18 to 87 years of age) for reasons that are not yet understood [1, 2]. Overall, the median age of kidney failure for ADTKD is estimated at ~50 [1]. ADTKD is an autosomal dominant disease and therefore runs in every generation of an affected family. At present, there are no specific treatments to prevent or cure the disease; however, research is being performed to find new therapies. General guidelines that aim to limit the progression of chronic kidney disease (CKD) should be followed in all ADTKD patients.

Most cases of ADTKD are caused by mutations in the *UMOD* gene (ADTKD-*UMOD*) and *MUC1* gene (ADTKD-*MUC1*). The other four subtypes do not present primarily as ADTKD [1–5]. The typical features of all six known subtypes are summarised in Table 1.

**Table 1:** The six different ADTKD subtypes and their typical features

ADTKD subtypes	Typical features
***UMOD***	*Gout prevalent (80%) and often early onset (typically adolescent to 3rd decade)* [2]
***MUC1***	*Gout less prevalent (25%) and later onset (typically 5th decade)* [2]
***REN***^**a**^	*Childhood anaemia, hypotension, early onset gout, hyperkalaemia, metabolic acidosis, low plasma renin* [6]
***HNF1β***	*Renal tract anomalies, monogenic diabetes, pancreatic hypoplasia, gout, female genital tract malformations, neurological features, deranged liver function tests, hypokalaemia and hypomagnesaemia*
***DNAJB11***	*Cystic kidney disease, vascular aneurysms/dissections, liver cysts* [4]*, and phenotypes are a hybrid of ADPKD and ADTKD*
***SEC61A1***	*Recurrent infections, abscess formation, leukopenia, neutropenia, congenital anaemia, bifid uvula, cleft palate and velopharyngeal insufficiency, pre-axial polydactyly, cognitive impairment, growth restriction, early onset gout*

^a^Mutations in signal peptide are the most common and give these features [6]

**Fig. 1:**
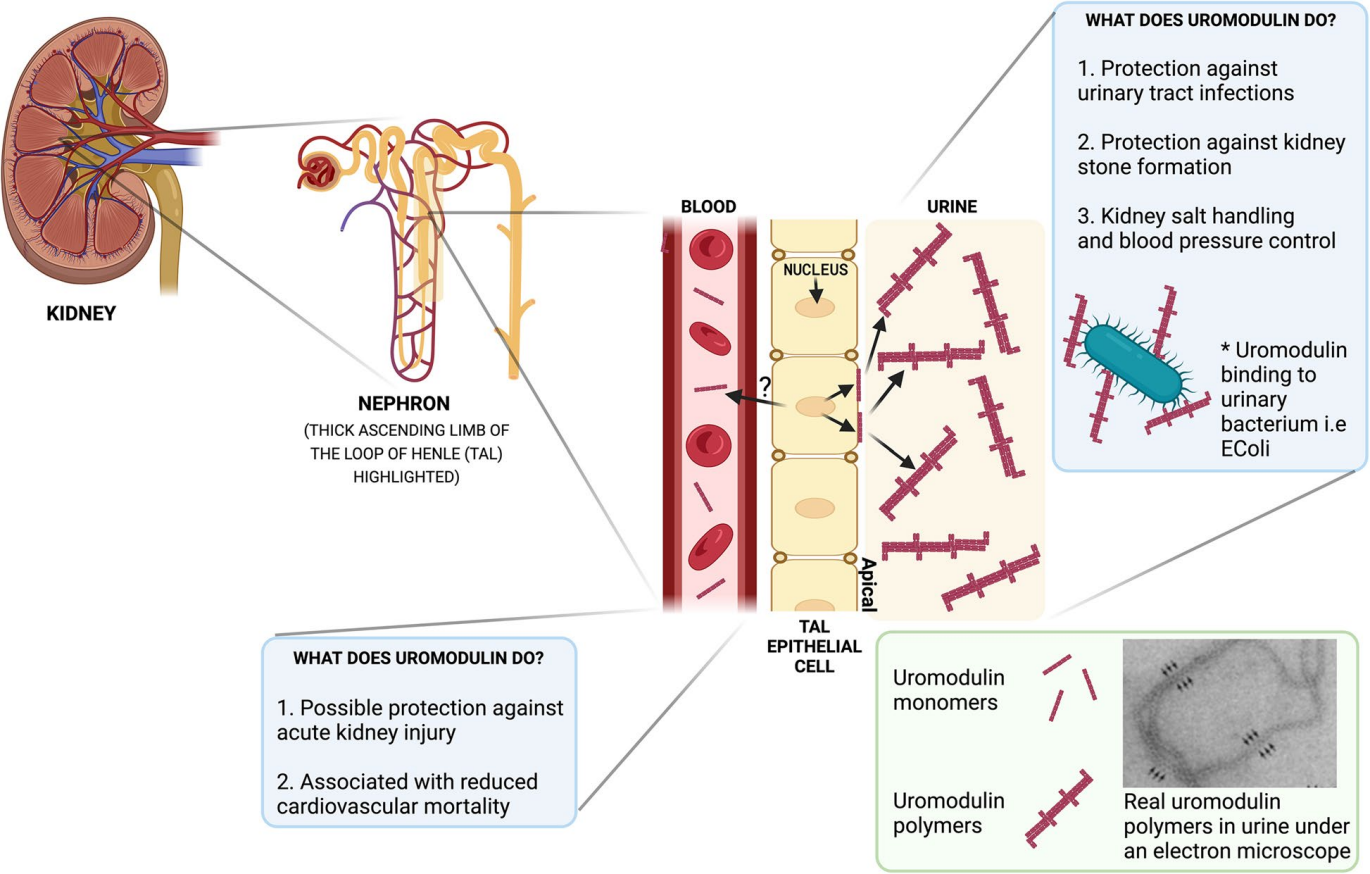
Uromodulin: where it is made and what it does. Uromodulin is made exclusively in the thick ascending limb of the loop of Henle (TAL) that is part of the nephron of which there are millions in the kidney. Uromodulin is excreted from the apical membrane of TAL cells into the urine in polymerised forms and is also found in the blood at a much lower concentration. Uromodulin has been shown to have multiple roles in both urine and blood with the most well described being its protection against urinary tract infections (UTIs). In the urine, the polymerised form binds to common urinary bacteria such as *E. coli* that can cause urinary tract infections. This prevents binding of these bacteria to cells of the bladder and subsequently encourages the bacteria to be removed from the body via the urinary tract to prevent infection

**Fig. 2:**
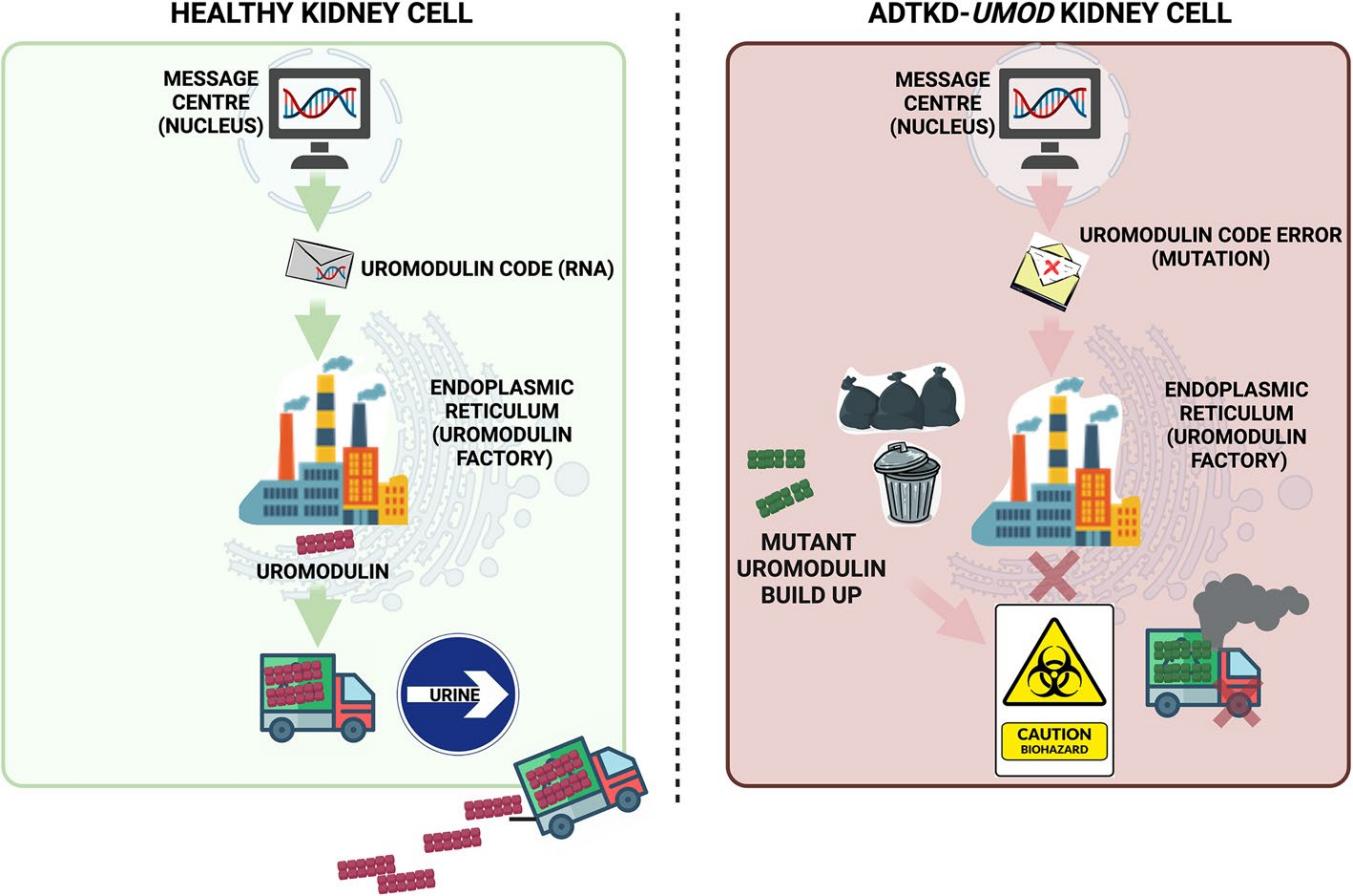
A simplified model of the disease processes underlying ADTKD-*UMOD* to enable patient discussions. In a healthy kidney cell (thick ascending limb of the loop of Henle (TAL) cell), the nucleus contains the correct ‘code’ of DNA (and RNA) for the endoplasmic reticulum (ER or ‘protein making factory’) to make the protein, uromodulin. Once this is made, it is transported to the urine. In the kidney (TAL) cell from a patient with ADTKD-*UMOD*, the *UMOD* gene (segment of DNA) has an error (mutation) in it. This code tells the ER to make a different form of uromodulin (a mutant form) that cannot escape the ER. It subsequently accumulates which is toxic to the cell and the cell subsequently becomes unwell. When many of these cells become unwell, they eventually trigger fibrosis or ‘scar tissue’ to develop within the kidney which leads to loss of kidney function over time

ADTKD is often under-recognised in the clinical setting for a variety of reasons, but the many different names used for the disease over recent years have been particularly confusing (Table 2). The true prevalence of the disease is therefore underestimated. ADTKD-*UMOD* has been shown to account for 2% of all end-stage kidney disease (ESKD) in one UK cohort, 1% of all CKD stage 3–5 and 9% of all inherited kidney diseases [7]. The *UMOD* gene encodes the protein uromodulin that is the most abundant protein in human urine and has been found to have many functions (Fig. 1). Within the kidney, the cellular consequences of *UMOD* mutations lead to endoplasmic reticulum (ER) stress which triggers interstitial nephritis (Fig. 2).

**Table 2:** Historical and up-to-date names for different types of ADTKD

Previous names for autosomal dominant tubulointerstitial kidney disease (ADTKD)	Modern nomenclature
*Medullary cystic kidney disease type 1*	*ADTKD-MUC1*
*Mucin-1 kidney disease*	*ADTKD-MUC1*
*Medullary cystic kidney disease type 2*	*ADTKD-UMOD*
*Familial juvenile hyperuricemia nephropathy type 1*	*ADTKD-UMOD*
*Uromodulin-associated kidney disease*	*ADTKD-UMOD*
*Renin-associated kidney disease*	*ADTKD-REN*
*Familial juvenile hyperuricemia nephropathy type 2*	*ADTKD-REN*

## Inheritance

**Fig. 3:**
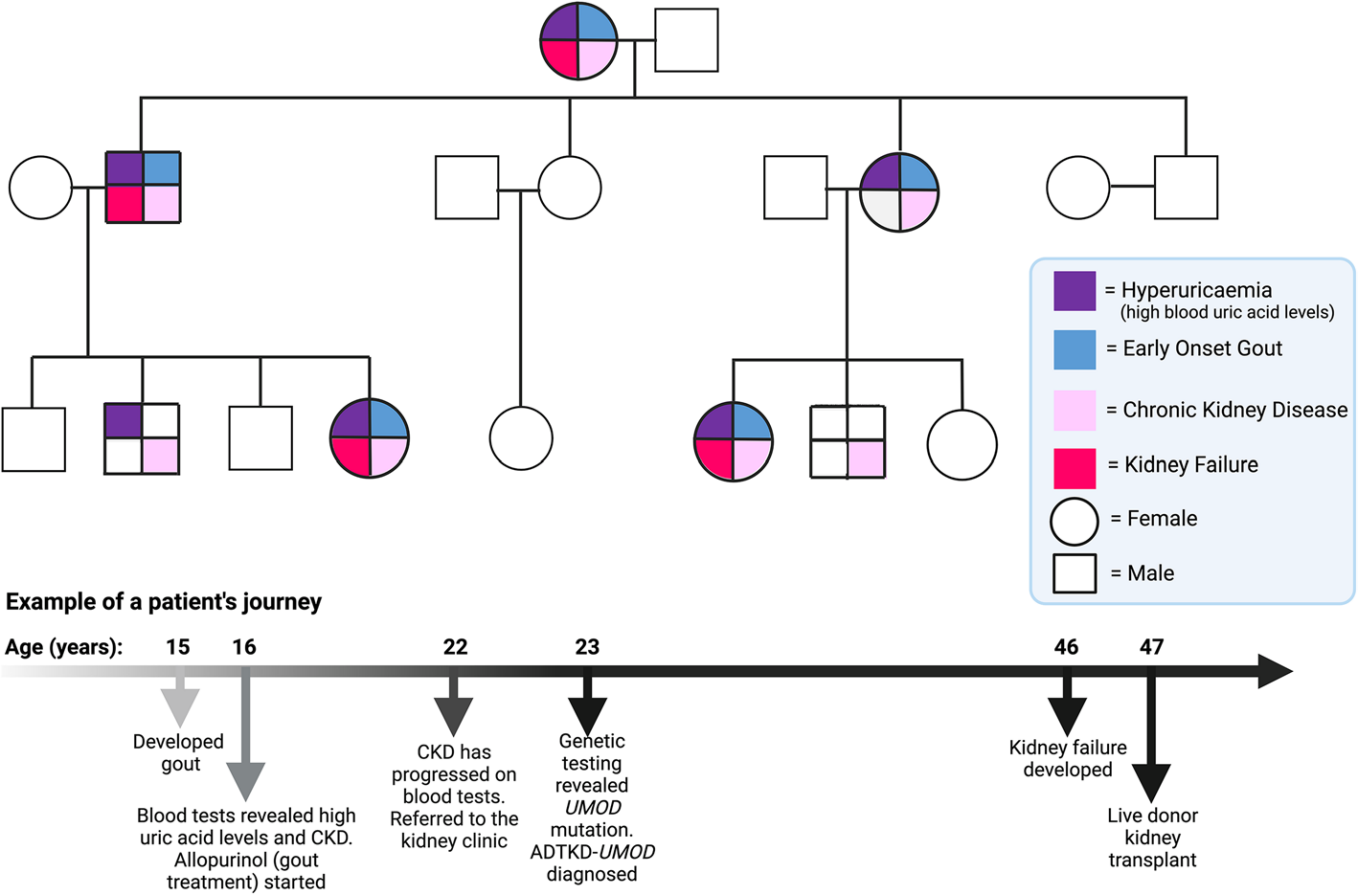
A typical family with ADTKD-*UMOD*. A pedigree diagram is shown illustrating the autosomal dominant pattern of inheritance and the phenotypic spectrum of disease. A typical timeline of a patient’s presentation and diagnosis is shown

ADTKD runs in families in an autosomal dominant fashion meaning that every generation may be affected, it does not skip generations and it affects both males and females equally. There is a 50% chance that the biological child of a person with ADTKD will inherit the disease (see Fig. 3 for an example of a typical family with ADTKD). However, de novo (or new) mutations can occur and in such cases there is not a positive family history of kidney disease in prior generations, but the mutation can subsequently be passed on to offspring (50% risk) [2].

## Symptoms and signs

ADTKD causes progressive damage in the kidneys exclusively, not any other organs; however, patients may experience a variety of symptoms that affect different parts of the body as a consequence of kidney failure itself. About three quarters of ADTKD-*UMOD* patients develop gout early in life that occurs *before* the onset of CKD, not as a consequence of it [6]. Gout is due to poor elimination of uric acid by the kidney [6].

Kidney failure patients typically develop tiredness and weakness. Some patients might also develop itch, leg cramps, restless legs and signs of fluid retention such as swollen legs and breathlessness. As kidney function worsens, blood pressure might become elevated; anaemia and hyperparathyroidism (overactive parathyroid gland) might develop. Medications are often started to prevent any negative consequences from these.

Decline in kidney function typically occurs in a steady and linear fashion and, for the majority of patients, is slow. Typically, the age that kidney failure is likely to develop can be predicted based on kidney function tests. An estimated 25% of ADTKD-*UMOD* patients have been shown to have kidney cysts [2, 6, 8], and some patients have symptoms and discrete signs of salt-wasting. Salt-wasting symptoms are typically ‘polyuria’ (passing large volumes or urine frequently or bed-wetting at night) and ‘polydipsia’ (excessive thirst) and occur early in the disease [9]. ADTKD does not recur after kidney transplantation [10].

## Diagnosis

**Fig. 4:**
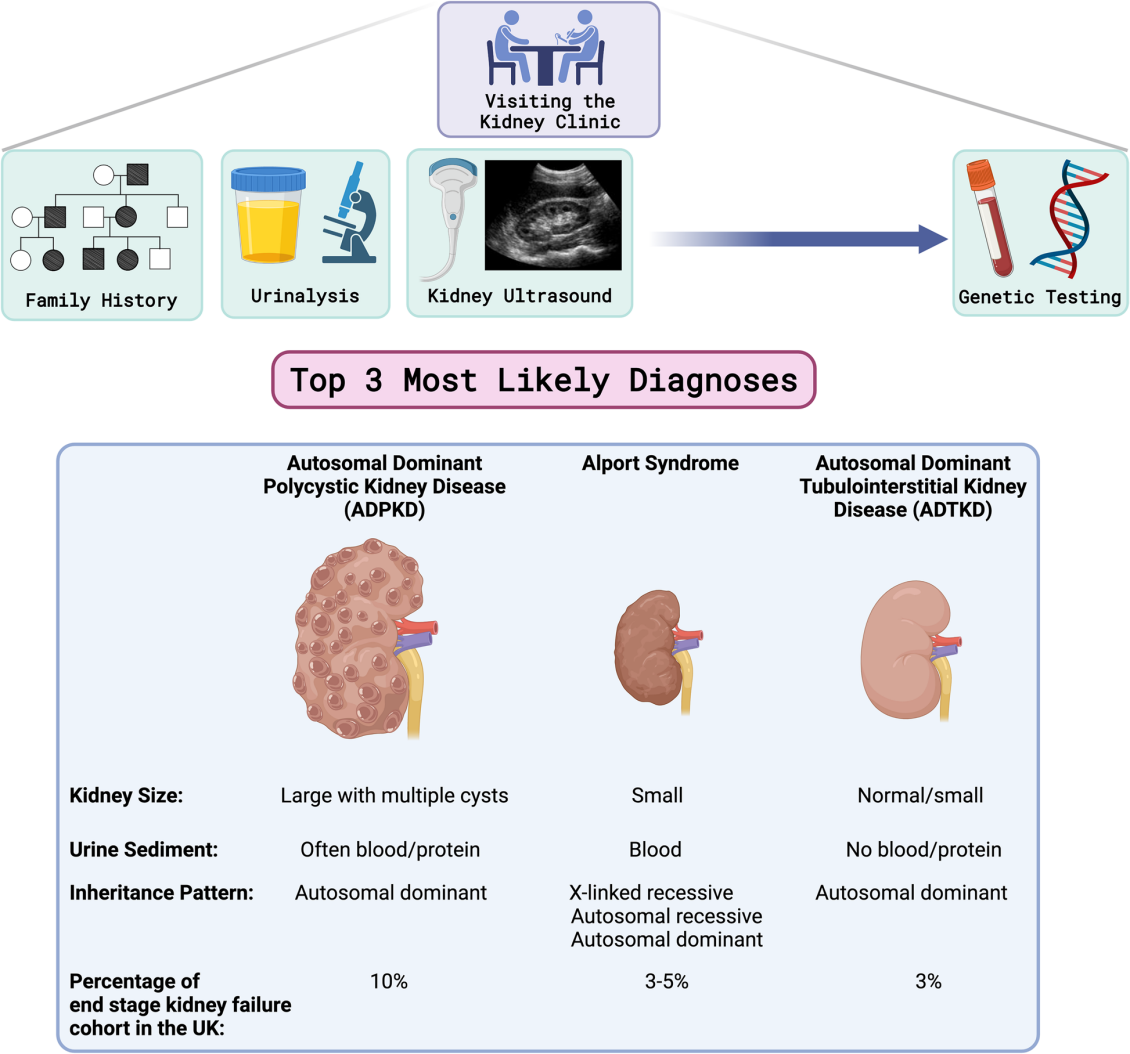
What happens when you visit the kidney clinic? The kidney clinic would allow a family history of kidney disease to be taken as well as blood and urine tests and kidney imaging. This would allow information to be gathered to determine the most likely genetic cause of inherited kidney disease. ADTKD typically gives a bland urine with no blood or protein in it and normal or small kidneys on imaging. There may be minor cystic changes within the kidney

Criteria that must be fulfilled to warrant a clinical suspicion of ADTKD are a positive autosomal dominant family history of CKD, absence of microscopic or visible blood in the urine, absence of significant amounts of urinary protein and normal or small sized kidneys (Fig. 4) [11]. A kidney biopsy is not necessary for diagnosis but classical biopsy features (interstitial fibrosis and tubular atrophy) or potential extra-renal manifestations (e.g. early onset gout) can help point towards the diagnosis. A definitive diagnosis of ADTKD requires identification of a mutation in one of the causative genes so genetic testing (usually in the form of a simple blood test) is the first-line approach. However, cases remain where no mutation has been identified in these genes, which is called ADTKD-*NOS* (*not otherwise specified*). A potential diagnosis of ADTKD should not be ignored in this instance, as new genetic causes still require identification [11]. Common alternative causes of kidney disease that might be diagnosed if a person does not have ADTKD are shown in Fig. 4.

Genetic testing ensures a definitive diagnosis and classification of ADTKD subtype. A genetic diagnosis will probably be necessary for inclusion in future clinical trials and allows for identification of affected family members. Genetically related family members will require genetic testing if they wish to be considered as a potential live donor for kidney transplantation. All patients and family members should be referred for genetic counselling. At present, there is no specific treatment for ADTKD, so learning of a diagnosis in childhood does not alter treatment; therefore, testing of children is not typically done but can be discussed with a specialist. Genetic testing is usually targeted to look for ADTKD-*UMOD* and *MUC1* first, and if this is negative, then other ADTKD genes should be tested. Testing can be performed via a nephrologist with a specialist interest in genetic kidney disease or a clinical geneticist. Of note, the most common mutation in *MUC1* cannot be identified using standard genetic testing (such as Sanger sequencing, exome and genome sequencing) but requires a more complex technique that may require DNA or other samples to be sent to a specialist centre that can do this [12].

## Treatments

There are currently no targeted therapies to cure or prevent ADTKD; however, the recommendations applied to the management of CKD should be followed, and a potential pharmacological treatment for ADTKD-*MUC1* which targets protein misfolding and ER stress might be on the horizon [13]. Cases of ADTKD-REN have been successfully treated with fludrocortisone to stabilise kidney function and improve serum electrolytes and acid base status [6]. Episodes of gout should be prevented using Allopurinol (or alternative urate lowering therapies) and low purine diet although this has not been proven to slow CKD progression. The angiotensin receptor blocker Losartan is the preferred treatment to lower blood pressure or reduce protein in the urine due to its additional effects on eliminating uric acid via the urine. Diuretics (water-eliminating treatment) should be used with caution in those with salt-wasting symptoms so that gout does not worsen and dehydration does not occur [14]. Transplantation, rather than dialysis, is the preferred method of renal replacement therapy for ADTKD patients as the disease does not recur in the transplanted kidney and outcomes are no worse than for those with other kidney diseases. Healthy lifestyle measures should ideally be followed (Table 3).

**Table 3:** Health advice for ADTKD patients

**What can I do for my health if I have ADTKD?**	
**Lifestyle**	
*Eat a low salt (sodium) diet with a lower protein content. The ‘DASH’ diet, a ‘plant-based’ diet and the ‘Mediterranean’ diet all have the most evidence for health benefits in kidney disease* [15–17]	
*Don’t smoke*	
*Exercise/move regularly in any way that suits you* [18]	
*Having a healthy weight will make you more likely to be eligible for a kidney transplant and prevent more rapid decline in your kidney function* [19]	
*Prioritise good quality sleep and limit stress in any way that works for you*	
*Check your blood pressure regularly (you can buy a home monitor or do this via your GP practice) and notify your doctor with the results at each visit or if the readings become higher*	
**Health care**	
*Attend your kidney clinic appointments to help you manage symptoms, cardiovascular risk and prepare you for kidney transplantation if you need it*	
**Family planning**	
*Discuss family planning with your kidney doctor early so that preparations can be made to ensure as safe a pregnancy as possible for mother and baby*	
*Preimplantation testing of embryos for ADTKD might be available so discuss this with your kidney doctor prior to pregnancy to see if it is possible (if you wish to consider this)*	

## Conclusions

ADTKD is a rare inherited kidney disease which leads to ESKD. Its early recognition and diagnosis will allow patients to receive a precise diagnosis and appropriate management. We anticipate new treatments will become available for this condition in the coming years. Explaining the diagnosis to physicians and patients is important given its insidious nature, pattern of inheritance and confusing nomenclature.

## Data Availability

Data sharing not applicable to this article as no datasets were generated or analysed during the current study.
